# Risk factors for peripartum hysterectomy among women with postpartum haemorrhage: analysis of data from the WOMAN trial

**DOI:** 10.1186/s12884-018-1829-7

**Published:** 2018-05-29

**Authors:** Sumaya Huque, Ian Roberts, Bukola Fawole, Rizwana Chaudhri, Sabaratnam Arulkumaran, Haleema Shakur-Still

**Affiliations:** 1 Clinical Trials Unit, London School of Hygiene and Tropical Medicine, Keppel Street, London, WC1E 7HT UK; 2 Department of Obstetrics and Gynaecology, National Institute of Maternal and Child Health, College of Medicine, University of Ibadan, Orita-Mefa, Ibadan, Nigeria; 3 Department of Obstetrics and Gynaecology, Rawalpindi Medical University, Rawalpindi, Pakistan; 4 St George’s University, Cranmer Terrace, London, SW17 0RE UK

**Keywords:** Peripartum hysterectomy, Postpartum haemorrhage, Placenta accreta, Caesarean section, Asia, Africa, Conceptual framework

## Abstract

**Background:**

Peripartum hysterectomy can cause significant morbidity and mortality. Most studies of peripartum hysterectomy are from high income countries. This cohort study examined risk factors for peripartum hysterectomy using data from Africa, Asia, Europe and the Americas.

**Methods:**

We used data from the World Maternal Antifibrinolytic (WOMAN) trial carried out in 193 hospitals in 21 countries. Peripartum hysterectomy was defined as hysterectomy within 6 weeks of delivery as a complication of postpartum haemorrhage. Univariable and multivariable random effects logistic regression models were used to analyse risk factors. A hierarchical conceptual framework guided our multivariable analysis.

**Results:**

Five percent of women had a hysterectomy (1020/20,017). Haemorrhage from placenta praevia/accreta carried a higher risk of hysterectomy (17%) than surgical trauma/tears (5%) and uterine atony (3%). The adjusted odds ratio (AOR) for hysterectomy in women with placenta praevia/accreta was 3.2 (95% CI: 2.7–3.8), compared to uterine atony. The risk of hysterectomy increased with maternal age. Caesarean section was associated with fourfold higher odds of hysterectomy than vaginal delivery (AOR 4.3, 95% CI: 3.6–5.0). Mothers in Asia had a higher hysterectomy incidence (7%) than mothers in Africa (5%) (AOR: 1.2, 95% CI: 0.9–1.7).

**Conclusions:**

Placenta praevia/accreta is associated with a higher risk of peripartum hysterectomy. Other risk factors for hysterectomy are advanced maternal age, caesarean section and giving birth in Asia.

## Background

Peripartum hysterectomy is performed at the time of delivery, or at any time from delivery to discharge from the same hospitalisation. The main indication for peripartum hysterectomy is severe uterine haemorrhage that cannot be controlled by conservative measures [1]. Peripartum hysterectomy is a “near-miss” maternal event - an intervention performed in life threatening obstetric situations to prevent death [2]. It results in the loss of fertility and is associated with significant maternal morbidity and mortality [3].

Worldwide, the rate of peripartum hysterectomy varies widely. In high income countries less than one in 1000 deliveries is complicated by peripartum hysterectomy [4–10], whereas in Nigeria [11] and Pakistan [12] the incidence is 4 and 11 per 1000 deliveries, respectively. The rate of emergency peripartum hysterectomy has been increasing over time [7–9, 13–15]. In USA, it increased by 12% between 1998 and 2003 [9] and by 15% between 1995 and 2007 [13].

The risk factors for peripartum hysterectomy are advanced maternal age, abnormal placentation, higher parity, and caesarean delivery in previous or current pregnancy [1, 16]. An increased risk of hysterectomy associated with placental pathologies and caesarean sections has been reported in several studies [5–7, 9, 10, 15, 17].

Individual studies on peripartum hysterectomy have small sample sizes, and the definition of peripartum hysterectomy varies across studies making comparisons difficult [16]. Systematic reviews often exclude studies conducted in underdeveloped nations [16, 18], or have an underrepresentation of women in poor countries [1]. In this study we used data from a large multinational clinical trial, in which most women were from Africa and Asia. The objective of this study was to i) determine the association between placenta praevia/accreta and the risk of emergency hysterectomy and ii) investigate the association between demographic and delivery-related risk factors and emergency hysterectomy.

## Methods

### Study design and data source

This is a cohort study using data from the World Maternal Antifibrinolytic (WOMAN) trial, which was a large, randomised, double blinded, placebo controlled trial conducted in 193 hospitals in 21 countries [19]. Women diagnosed with postpartum haemorrhage (PPH) were randomised to receive tranexamic acid or placebo. Baseline data were recorded in an entry form prior to randomisation and outcome data collected at death, discharge from hospital or 42 days following randomisation, whichever occurred first [20]. All women with a completed outcome form were included in our cohort study, irrespective of the trial arm they were randomised to.

Our study outcome was defined as hysterectomy performed before discharge or within 6 weeks of delivery. The primary exposure of interest was placenta praevia/accreta as cause of haemorrhage. Other risk factors evaluated in this study include, maternal age, geographic region, delivery in study hospital, administration of prophylactic uterotonics, type of delivery, full delivery of placenta, systolic blood pressure (SBP), estimated volume of blood loss, and clinical signs of haemodynamic instability. Countries were categorised into three geographic regions as follows: Africa - Burkina Faso, Cameroon, Cote d’Ivoire, Democratic Republic of Congo, Egypt, Ethiopia, Ghana, Kenya, Nigeria, Sudan, Tanzania, Uganda and Zambia; Asia - Bangladesh, Nepal, Pakistan, Papua New Guinea; Europe and the Americas - Albania, Colombia, Jamaica and United Kingdom.

### Statistical analyses

Stata 14 was used for all statistical analyses [21]. We used frequencies and percentages to describe the characteristics of the study population. For variables with more than 1% missing data (uterotonics administered prophylactically), we explored to check if missingness was random. To account for clustering of subjects at the hospital level, we fitted random effects logistic regression models adjusting for hospital. We included age as a forced variable, a priori, for all univariable analyses.

**Fig. 1:**
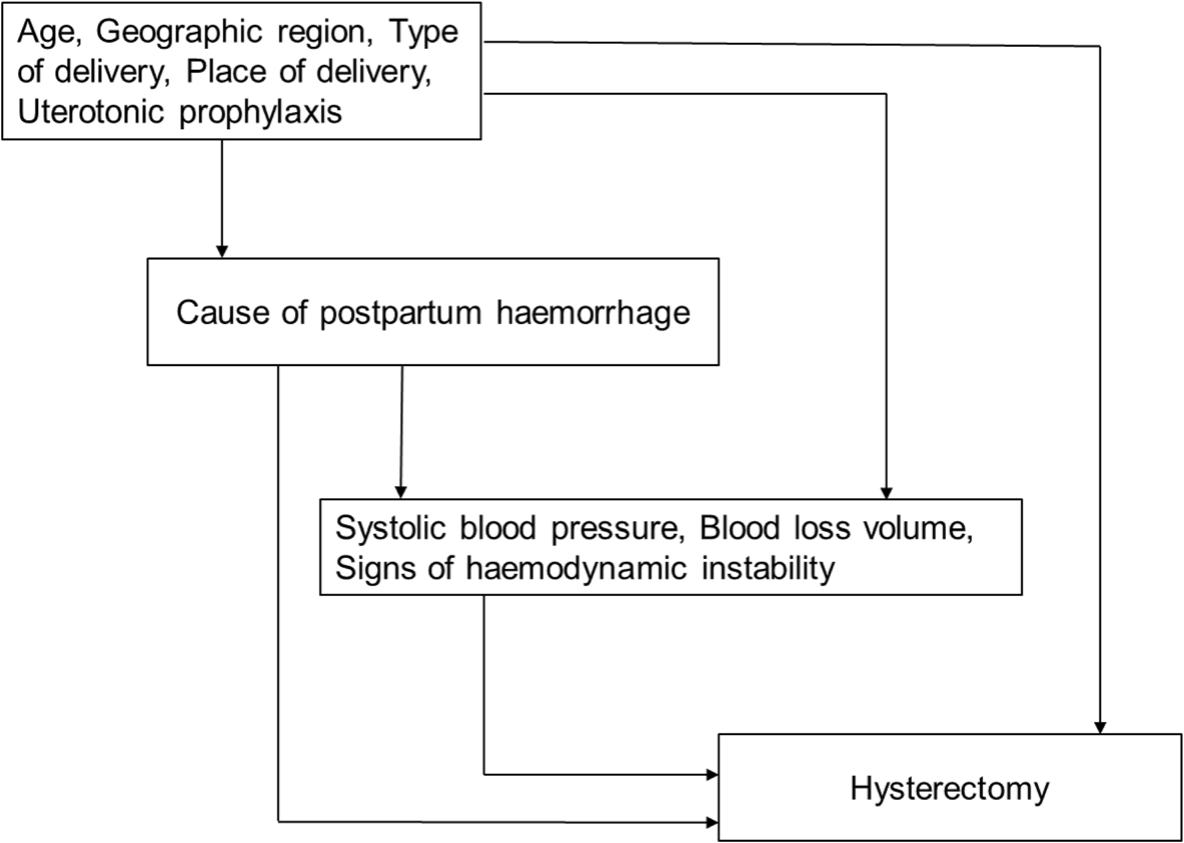
Hierarchical conceptual framework of risk factors for hysterectomy in women with postpartum haemorrhage

Drawing on prior knowledge, we hypothesized variables that might be confounders and those that might be in the causal pathway between our primary exposure and outcome [22]. We excluded full delivery of placenta from multivariable models since any placental pathology would affect its delivery. Variables reflecting haemodynamic status such as blood pressure and blood loss volume were identified as mediators, because cause of haemorrhage can influence these variables and subsequently the need for hysterectomy. The hierarchical conceptual framework shown in Fig. 1 guided our analysis.

A forward approach was used to build multivariable models. Exposure variables in model 1 were cause of PPH, age, geographic region, delivery in study hospital, administration of prophylactic uterotonics and type of delivery. Model 2 included variables in model 1 plus systolic blood pressure, estimated volume of blood loss and clinical signs of haemodynamic instability. For each hierarchy, the association between cause of haemorrhage and hysterectomy was adjusted by adding variables one at a time, based on the magnitude of effect in crude analysis. As each variable was added to the model, we assessed for collinearity. After fitting a preliminary final model, negative confounding was explored by including variables that were initially excluded. A variable was a negative confounder if it did not show an association at univariable analysis, but became associated with the outcome at multivariable analysis (*p* < 0.05). Once a full model was identified, for each hierarchy, risk factors were evaluated statistically using the likelihood ratio test.

## Results

Table 1 shows characteristics of the 20,021 study participants. The mean maternal age was 28 years (standard deviation: 6 years). Sixty four percent of mothers were from Africa and 31% from Asia. The most common cause of postpartum haemorrhage was uterine atony (64%), followed by surgical trauma (18%) and placenta praevia/accreta (9%). Most mothers (88%) gave birth at study hospitals, whereas 12% gave birth in other settings and were referred to study sites after PPH onset. Seventy one percent of mothers delivered vaginally and 29% had caesarean sections.

**Table 1:** Baseline characteristics of women with postpartum haemorrhage (*N* = 20,021)

Variable	Number	Percent
Cause of haemorrhage		
Uterine atony	12,761	63.7
Surgical trauma/tears	3681	18.4
Placenta praevia/accreta	1875	9.4
Other/Unknown	1700	8.5
Missing	4	0.02
Age (years)		
< 20	1021	5.1
20–29	10,410	52.0
30–39	7902	39.5
≥40	681	3.4
Missing	7	0.03
Mean (SD)	28.3 (5.7)	
Geographic Region		
Africa	12,718	63.5
Asia	6173	30.8
Europe and Americas	1130	5.6
Delivery in study hospital		
Yes	17,590	87.9
No	2428	12.1
Missing	3	0.01
Uterotonic prophylaxis given		
Yes	19,268	96.2
No	269	1.3
Missing	484	2.4
Type of delivery		
Vaginal	14,191	70.9
Caesarean	5825	29.1
Missing	5	0.02
Full delivery of placenta		
Yes	18,067	90.2
No	1951	9.7
Missing	3	0.01
Systolic Blood Pressure (mmHg)		
≤70	1532	7.7
71–90	5927	29.6
> 90	12,557	62.7
Missing	5	0.02
Mean (SD)	100.8 (22.7)	
Estimated volume of blood loss (mL)		
≤ 1000	10,403	52.0
1001–1500	5704	28.5
> 1500	3911	19.5
Missing	3	0.01
Mean (SD)	1226.4 (617.8)	
Clinical signs of haemodynamic instability		
No	8194	40.9
Yes	11,826	59.1
Missing	1	< 0.01

There were 484 women with missing data for uterotonic prophylaxis. They had a higher risk of hysterectomy (odds ratio 1.5, 95% CI: 1.0–2.1, *p* = 0.04) compared to women with data for uterotonic prophylaxis. Among the women with missing data for uterotonic prophylaxis, 468 (97%) gave birth in a setting other than the study sites. The remaining exposure variables had less than 1 % missing data. Four women had missing outcome data.

The risk of peripartum hysterectomy within 6 weeks of PPH diagnosis was 5% (1020/20,017). The risk of hysterectomy for uterine atony, surgical trauma/tears and placenta praevia/accreta was three, five and 17 %, respectively (Table 2). The incidence of hysterectomy was 1% in mothers under 20 years and 13% in mothers who were 40 and above. The risk of hysterectomy varied by geographic region: 7% in Asia and 5% in Africa. Women having caesarean sections had a higher risk of hysterectomy (11%) than women who delivered vaginally (3%). The age-adjusted odds ratio for placenta praevia/accreta and hysterectomy was 4.9 (95% CI: 4.1–5.8) compared to uterine atony.

**Table 2:** Univariable analysis of risk factors for hysterectomy in women with postpartum haemorrhage

Variable	Total	n(%) of women who had hysterectomy	Age-adjusted odds ratio (95% CI)^a^	*P*-value†
Cause of haemorrhage				
Uterine atony	12,759	421 (3.3)	1	< 0.001
Surgical trauma/tears	3681	181 (4.9)	1.49 (1.24–1.80)	
Placenta praevia/accreta	1874	326 (17.4)	4.87 (4.11–5.77)	
Other/Unknown	1699	90 (5.3)	1.40 (1.10–1.80)	
Age (years)				
< 20	1021	12 (1.2)	1	< 0.001
20–29	10,408	306 (2.9)	2.15 (1.20–3.86)	< 0.001^ǂ^
30–39	7900	615 (7.8)	5.98 (3.34–10.70)	
≥40	681	87 (12.8)	11.73 (6.30–21.85)	
Geographic region				
Africa	12,714	570 (4.5)	1	< 0.001
Asia	6173	420 (6.8)	1.67 (1.20–2.34)	
Europe and Americas	1130	30 (2.7)	0.53 (0.26–1.08)	
Delivery in study hospital				
Yes	17,586	868 (4.9)	1	0.77
No	2428	152 (6.3)	0.97 (0.80–1.18)	
Uterotonic prophylaxis given				
Yes	19,264	940 (4.9)	1	< 0.001
No	269	40(14.9)	2.65 (1.80–3.88)	
Type of delivery				
Vaginal	14,188	357 (2.5)	1	< 0.001
Caesarean	5824	661 (11.4)	4.50 (3.89–5.21)	
Full delivery of placenta				
Yes	18, 064	796 (4.4)	1	< 0.001
No	1950	223 (11.4)	2.24 (1.89–2.66)	
Systolic blood pressure (mmHg)				
≤70	1532	210 (13.7)	6.40 (5.25–7.81)	< 0.001
71–90	5925	453 (7.7)	3.02 (2.59–3.53)	< 0.001^ǂ^
> 90	12,555	357 (2.8)	1	
Estimated volume of blood loss (mL)				
≤ 1000	10,400	86 (0.8)	1	< 0.001
1001–1500	5703	238 (4.2)	5.35 (4.13–6.93)	< 0.001^ǂ^
> 1500	3911	696 (17.8)	32.02 (24.97–41.05)	
Clinical signs of haemodynamic instability				
No	8194	128 (1.6)	1	< 0.001
Yes	11,822	891 (7.5)	7.35 (5.96–9.06)	

^a^CI, confidence interval

†*P*-values obtained from likelihood ratio test

ǂLikelihood ratio test for linear trend

The timing of hysterectomy differed by cause of bleeding. The median time between PPH diagnosis and hysterectomy was 0.6 h for placenta praevia/accreta, compared to ≥1.3 h for uterine atony, surgical trauma/tears and other/unknown causes of PPH. The median time between PPH diagnosis and hysterectomy was shorter in Asia (0.8 h) than in Africa (1.6 h). The time from PPH to hysterectomy in Asia was 0.5 h for placenta praevia/accreta, 0.9 h for surgical trauma/tears and 1.0 h for uterine atony. The time from PPH to hysterectomy in Africa was 1.3 h for placenta praevia/accreta, 1.9 h for surgical trauma and 1.8 h for uterine atony.

Table 3 shows adjusted odds ratios (AOR) for the association between exposure variables and hysterectomy. Placenta praevia/accreta was associated with a threefold higher risk of peripartum hysterectomy than uterine atony (AOR 3.2, 95% CI: 2.7–3.8). The AORs for hysterectomy and maternal age was 1.6 (95% CI: 0.9–2.9) in 20–29 years, 4.0 (95% CI: 2.2–7.1) in 30–39 years and 7.6 (95% CI: 4.0–14.3) in ≥40 years compared to < 20 years. Mothers in Asia had a 23% higher risk of hysterectomy than mothers in Africa, although the estimate was imprecise in model 1. After adjusting for variables in model 2 the odds ratio for hysterectomy in women giving birth in Asia was 2.2 (95% CI: 1.5–3.1) compared to women giving birth in Africa. The odds of hysterectomy was fourfold higher in women having caesarean sections than in women delivering vaginally (AOR 4.3, 95% CI: 3.6–5.0). There was strong evidence that the association between type of delivery and hysterectomy varied by cause of haemorrhage (*p*-value for interaction < 0.001). The adjusted odds ratio for caesarean section and hysterectomy was 10.0 (95% CI: 6.2–16.0) in women with placenta praevia/accreta, 3.4 (95% CI: 2.8–4.3) in women with uterine atony and 3.7 (95% CI: 2.6–5.2) in women with surgical trauma/tears. Place of delivery was a negative confounder, that was not associated with hysterectomy on univariable analysis but became associated on multivariable analysis. Women who delivered somewhere other than study hospitals were one and half times more likely to undergo hysterectomy (AOR 1.6, 95% CI: 1.3–2.1).

**Table 3:** Adjusted odds ratios for risk factors of hysterectomy in women with postpartum haemorrhage

Variable	Model 1^a^		Model 2^b^	
	AOR (95% CI)^c^	*P*-value^†^	AOR (95% CI)^c^	*P*-value^†^
Cause of haemorrhage				
Uterine atony	1	< 0.001	1	< 0.001
Surgical trauma/tears	1.42 (1.16–1.73)		1.21 (0.98–1.50)	
Placenta praevia/accreta	3.17 (2.66–3.79)		2.25 (1.85–2.74)	
Other/Unknown	1.34 (1.03–1.74)		0.97 (0.73–1.28)	
Age (years)				
< 20	1	< 0.001	1	< 0.001
20–29	1.62 (0.90–2.93)		1.47 (0.80–2.72)	
30–39	3.96 (2.20–7.14)		3.33 (1.82–6.12)	
≥40	7.59 (4.03–14.29)		5.89 (3.04–11.38)	
Geographic region				
Africa	1	0.01	1	< 0.001
Asia	1.23 (0.91–1.66)		2.16 (1.52–3.08)	
Europe and Americas	0.45 (0.23–0.89)		0.69 (0.32–1.47)	
Delivery in study hospital				
Yes	1	< 0.001	1	0.06
No	1.60 (1.25–2.07)		0.77 (0.59–1.01)	
Uterotonic prophylaxis given				
Yes	1	< 0.001	1	< 0.001
No	2.68 (1.77–4.06)		2.57 (1.62–4.09)	
Type of delivery				
Vaginal	1	< 0.001	1	< 0.001
Caesarean	4.26 (3.60–5.04)		2.32 (1.92–2.80)	
Systolic blood pressure (mmHg)				
≤70			2.26 (1.79–2.87)	< 0.001
71–90			1.68 (1.40–2.01)	
> 90			1	
Estimated volume of blood loss (mL)				
≤ 1000			1	< 0.001
1001–1500			2.89 (2.19–3.83)	
> 1500			12.88 (9.78–16.95)	
Clinical signs of haemodynamic instability				
No			1	< 0.001
Yes			3.50 (2.74–4.47)	

^a^Model 1: cause of haemorrhage, age, geographic region, delivery in study hospital, uterotonic prophylaxis given and type of delivery

^b^Model 2: model 1 plus systolic blood pressure, estimated volume of blood loss and clinical signs of haemodynamic instability

^c^AOR: Adjusted odds ratio; CI: Confidence interval

†*P*-values obtained from likelihood ratio test

In our cohort of 20,021 women, 483 (2%) died. Mortality from postpartum haemorrhage was 3% (375/12,718) in Africa, 2% in Asia (106/6173) and 0.2% (2/1130) in Europe and the Americas. Out of 1020 mothers who had hysterectomies, 163 died. The overall case fatality rate was 16 per 100 hysterectomies. Death rate among hysterectomy cases was higher in Africa than in Asia, and Europe and the Americas: 20 compared to 11 and 7 per 100 hysterectomies, respectively.

The following sensitivity analyses were carried out and they did not change the results of the main analysis: i) Women who died before having a hysterectomy were excluded from multivariable analysis, ii) Women with SBP ≤30 mmHg were excluded from final models, and iii) Sensitivity analysis for missing data for uterotonic prophylaxis was carried out by assessing odds ratios in extreme situations, when all women with missing data received uterotonic prophylaxis and vice versa.

## Discussion

Our study shows that haemorrhage from placenta praevia/accreta increases the risk of peripartum hysterectomy. Other risk factors for hysterectomy are advanced maternal age, having a caesarean section and giving birth in Asia.

Our results should be interpreted in light of the study strengths and limitations. Data collection was complete and there was minimal missing data. The prospective collection of exposure data minimised recall and observer bias. The outcome, hysterectomy was an objective ascertainable variable unlikely to be misclassified. Measurement error may have occurred with age, SBP and volume of blood loss. In areas without legally enforced birth registration (e.g. some African and Asian countries), it can be argued that some women did not know their age reliably. Nonetheless, any misclassification was likely to be non-differential with minimal effect on result interpretation.

This study had a large sample size. Women were recruited from Africa and Asia where the incidence of PPH is high, thus increasing the study’s statistical power. Our results are generalizable to women with postpartum haemorrhage in low and middle income countries. However it is worth mentioning that the study sites were selected based on their ability to conduct a trial and the level of obstetric service available. Women, who deliver in hospitals involved in a multinational clinical trial, or are referred to them, may not be representative of all women who have PPH. Especially in regards to Africa and Asia, these women may be better off socioeconomically and medically, and our study may have excluded rural and poor women in these regions.

For multivariable analysis, we used sequential modelling of groups of risk factors according to their occurrence. This prevented adjusting for potential causal intermediates and allowed separation of confounding and mediating factors. We also adjusted for clustering at the hospital level. However, unmeasured confounding may have resulted from lack of data on risk factors such as parity and prior caesarean section. A further limitation of our study was the reporting of placenta praevia and accreta as one variable; it would have been informative to investigate each separately.

Our finding, of an increased risk of hysterectomy with abnormal placentation is consistent with other literature [1, 5, 7, 15, 16, 23]. In placenta accreta, the placental tissue invades the myometrium. After birth, it remains attached firmly to the uterine wall causing severe blood loss. The time to attempt conservative management is limited in placenta accreta, and obstetricians may proceed to hysterectomy directly. This is reflected by a shorter time gap between PPH diagnosis and hysterectomy in women with placenta praevia/accreta compared to other causes of haemorrhage. This pattern persisted after exclusion of women who were referred to study hospitals from elsewhere, to account for travel time. Some obstetricians opt for a conservative mode of treatment in abnormal placentation and leave the placenta in situ; however, this can lead to sepsis and secondary haemorrhage and ultimately hysterectomy [24]. Furthermore, haemostatic interventions in placenta praevia/accreta are associated with adverse effects. Radiological balloons, embolization and B-Lynch sutures can cause thrombosis, ischemia and neurological complications [24]. Risk factors for placenta accreta include previous caesarean section, other previous uterine surgery and advanced maternal age [25]. Caesarean section results in scarring of the uterus, which in later pregnancies predisposes to abnormal placentation [26]. The main indication for peripartum hysterectomy has shifted in recent decades from uterine atony to placenta accreta, in conjunction with a rise in caesarean delivery rates [27].

After adjusting for confounders, we found a higher risk of hysterectomy in older mothers. A lower threshold for hysterectomy in older women, who are likely to have more children, may explain this finding. We also found that having a caesarean section increases mothers’ risk of hysterectomy. One possible explanation for this observation is a lower threshold for hysterectomy during caesarean section, when the patient is already in the operating room and the uterus is readily accessible [18, 28]. In contrast, practitioners may be more likely to try other methods to control haemorrhage in vaginal deliveries because the uterus is not readily available for removal. Another explanation is that mothers undergo prenatal scanning with ultrasonography and magnetic resonance imaging [29]. Women with a prenatal diagnosis of placenta praevia/accreta are selected for caesarean section and may inevitably require a hysterectomy. In our cohort, the risk of hysterectomy associated with caesarean section varied by cause of haemorrhage. We found evidence that women with placenta praevia/accreta had a higher risk of hysterectomy associated with caesarean section than women with other causes of PPH.

Our results showed that mothers in Asia have a higher risk of hysterectomy than mothers in Africa. Caesarean section, which is a risk factor for hysterectomy, is carried out more frequently in Asia than in Africa [30]. The low rates of caesarean delivery in sub-Saharan Africa are presumably due to low levels of access to emergency surgical care, lack of skilled workers and poor infrastructure [31]. In the WOMAN trial, 25% of the deliveries in Africa were caesarean sections, compared to 37% in Asia and 32% in Europe and Americas. However, after adjusting for type of delivery and other potential confounders, the risk of hysterectomy remained high in Asia compared to other regions. This finding may be due to regional differences in obstetric practices and management of PPH. Although the risk of hysterectomy was higher in Asia than in Africa, mortality was lower (3% in Africa and 2% in Asia). Analysis of maternal mortality using country specific data found higher number of maternal deaths in Africa than in Asia [32]. It is possible that by carrying out hysterectomies promptly more lives are saved in Asia, and fewer mothers die from postpartum haemorrhage.

## Conclusions

Our study found a strong association between placenta praevia/accreta and peripartum hysterectomy. Other significant risk factors include advanced maternal age, caesarean section and giving birth in Asia. Preventing avoidable causes of placenta accreta such as unnecessary caesarean sections is recommended. Further research is warranted to understand how maternal demographics and local culture affect decision making in emergency obstetric situations.

## Abbreviations

AOR: Adjusted odds ratio
CI: Confidence interval
PPH: Postpartum haemorrhage
SBP: Systolic blood pressure
SD: Standard deviation
WOMAN: World Maternal Antifibrinolytic
